# Physiology, for the Use of Elementary Schools

**Published:** 1839-10

**Authors:** 


Art. IV.—*Human Physiology, for the Use of Elementary Schools.* By C. A. Lee, m.d. late Professor of Materia Medica in the University of New York. *Second Edition.—New York,* 1839. Post 8vo, pp. 336.

In our January Number we noticed a small work by Dr. Hayward, of Boston, written on somewhat the same plan as the present, and with the same praiseworthy view of rendering the science of physiology intelligible to general students. Dr. Lee's little volume, as will be seen by its title, goes even a step lower, and professes to instruct children, the boys and girls of elementary schools. We refer to our former notice for our views on this important subject; but, as we think the thing cannot be too strongly impressed upon the mind, we here repeat our conviction of the expediency, we may say the necessity, of having this branch of study introduced to our British schools. It would appear from the volumes of Dr. Hayward and Dr. Lee, that our American brethren have taken the lead of us in this as in many other things. The general plan of Dr. Lee's little book is excellent; the descriptions being in general clear and simple—in some cases we think not sufficiently explicit,—and every difficulty smoothed by copious illustration from woodcuts. These woodcuts, by the way, are, like all those in American books we have yet seen, stiff and harsh, and very inferior to the present state of the art in England. As we observe that Dr. Lee is about to publish a *New Medical Flora of North America*, illustrated by woodcuts—a work which is much needed and which could not be in better hands—we beg to call his attention to this subject of wood-engraving, in order that the excellencies of the author may not be impaired by the demerits of the artist.

